# Rho/ROCK Signaling Pathway in Kidney Diseases: Mechanisms and Therapeutic Perspectives

**DOI:** 10.3390/biomedicines14030621

**Published:** 2026-03-10

**Authors:** Wei Xiong, Daojia Miao, Zongchen Hou, Xiaoping Zhang, Zhiyong Xiong

**Affiliations:** 1 Department of Nephrology, Union Hospital, Tongji Medical College, Huazhong University of Science and Technology, Wuhan 430022, China; lucyjane520@126.com; 2 Department of Urology, Union Hospital, Tongji Medical College, Huazhong University of Science and Technology, Wuhan 430022, China; miaodj@hust.edu.cn (D.M.); zongchenhou@163.com (Z.H.)

**Keywords:** Rho, G protein, Rho associated coiled coil forming protein kinase (ROCK), kidney diseases, signaling pathway

## Abstract

Rho GTPases are a group of guanosine triphosphate (GTP)-binding proteins with a relative molecular weight of about 20–30 kD, and 22 different Rho GTPases have been identified in mammalian cells, among which RhoA, Rac1 and Cdc42 are the most well-studied. Rho-associated coiled coil forming protein kinase (ROCK) is the most well-researched downstream effector of Rho GTPases. The Rho/ROCK signaling pathway widely participates in the reorganization of the cytoskeleton through cascade phosphorylation/dephosphorylation reactions and modulates cellular biological behaviors including cell adhesion, migration and phenotypic transformation. Abnormal activation of the Rho/ROCK signaling pathway is closely associated with the occurrence and progression of acute kidney injury, diabetic nephropathy, hypertension-related nephropathy and chronic allograft nephropathy, which contributes to podocyte injury, renal tubular epithelial-to-mesenchymal transition (EMT), mesangial cell proliferation and inflammatory infiltration in the kidney. This review focuses on the research progress and regulatory mechanisms of the Rho/ROCK signaling pathway in the above four major kidney diseases and discusses the therapeutic potential of targeting this pathway for kidney disease treatment, aiming to provide new insights for elucidating the pathogenesis of kidney diseases and developing novel therapeutic strategies.

## 1. Introduction

### 1.1. Basic Characteristics and Regulation of Rho GTPases

G protein is the most classical signal molecule in cell signal transduction pathway, which includes the following two types: one is heterotrimeric G protein coupled with membrane receptor mediating transmembrane signal transduction of most cytokines, growth factors and vasoactive substances; the other type is called small G protein, which is a single subunit protein with molecular weight between 20 and 30 kD. According to the similarity of sequence homology, small G proteins can be divided into the following five subfamilies: Ras, Rab, Rho, Arf and Ran. In 1985, Rho was first cloned as a Ras homolog [1]. There are 22 different Rho proteins or Rho GTPases in mammalian cells, such as RhoA, RhoB, RhoC, Rac1 and Rac2, Cdc42, RhoG, RhoD and RhoE. Among them, RhoA, Rac1 and Cdc42 are the most well-studied [2]. Rho proteins are mostly distributed in cells with contractile motor function, such as cardiomyocytes, smooth muscle cells, tumor cells with invasive growth ability, fibroblasts, and glomerular mesangial cells [3].

**Figure 1:**
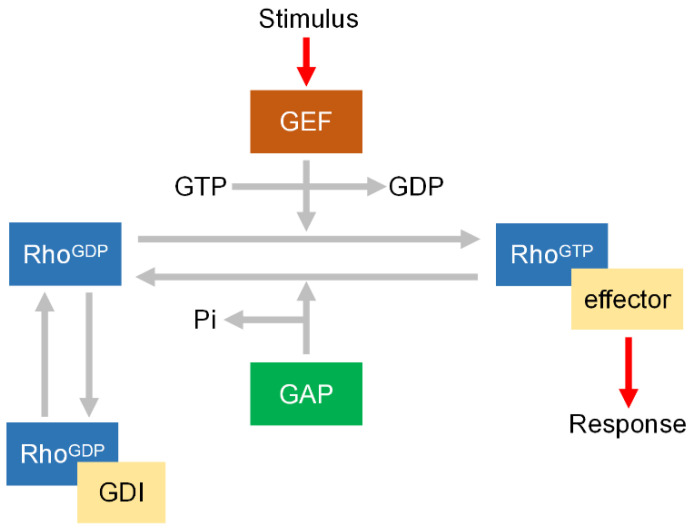
Schematic representation of the classical Rho GTPase regulatory cycle. Growth factor receptors or integrins can recruit GEF and Rho to the membrane, leading to the activation of Rho. GAP catalyzes the hydrolysis of Rho-GTP to Rho-GDP. GDI binds to Rho-GDP keep Rho remain inactive state. GTP, guanosine triphosphate; GEF, guanine nucleotide exchange factor; GAP, GTPase activating protein; GDI, guanine nucleotide dissociation inhibitor; GDP, guanosine diphosphate.

Rho proteins contain highly conserved GDP/GTP binding region and GTPase active region like all small G proteins in structure. They play an important molecular switching role in the way of inactive form-binding GDP and active form-binding GTP. Rho proteins have low intrinsic GTPase activity and have evolved a set of regulatory factors specifically regulating their activity in cells, including guanine nucleotide exchange factors (GEFs), GTPase activating proteins (GAPs), and guanine nucleotide dissociation inhibitors (GDIs) [4,5]. GAPs can promote the inactivation of active Rho GTPase. The principle is that once combined with Rho proteins, GAPs significantly increase the intrinsic GTPase activity of Rho GTPase, catalyze the dephosphorylation of GTP into an inactive GDP binding state, and then free back to the cytoplasm. GDIs maintain the Rho protein inactive by sequestering it away from the plasma membrane. GEFs are the main factors accelerating Rho activation by promoting the separation of Rho from GDP and then binding with GTP. Its activity is regulated by a variety of signal pathways, such as protein kinase, phosphatidylinositol kinase [6]. There is also evidence that Rho proteins are activated by Gq and Gi families of G proteins, but the specific mechanism remains unclear [7,8]. The activated Rho proteins are translocated from cytoplasm to cell membrane through the interaction of its carboxyl end with a specific target region [9,10]. The classical regulatory cycle of Rho GTPases is shown in Figure 1.

Rho proteins and its activity regulators are affected by a variety of extracellular stimulus signals, including membrane receptor signals and non-membrane receptor signals [9]. The former includes G protein-coupled growth factors and cytokine receptors, and the latter includes high glucose signals, phospholipase C, lysophosphatidic acid (LPA), etc. There are also many downstream effector molecules of Rho proteins such as Rho-associated coiled coil forming protein kinase (ROCK), mDia, Rhophillin, Rhotekin, citron and protein kinase N [11,12].

### 1.2. The Role of Rho Signaling in the Kidney

Rho GTPases are widely expressed in all major renal cell types, including podocytes, mesangial cells, proximal tubular epithelial cells and glomerular endothelial cells, and their activity is tightly controlled by cell-type specific GEFs, GAPs and GDIs. Core Rho GTPases (Cdc42, Rac1, and RhoA) and their regulatory factors form a complex network in renal cells, and the downstream effector layer includes ROCK1/ROCK2, PAK4, N-WASP, Synaptopodin and other actin-remodeling proteins. This network is essential for maintaining normal renal physiological functions, such as glomerular filtration barrier integrity, renal microvascular tone regulation, tubular epithelial cell polarity and adhesion [9].

**Figure 2:**
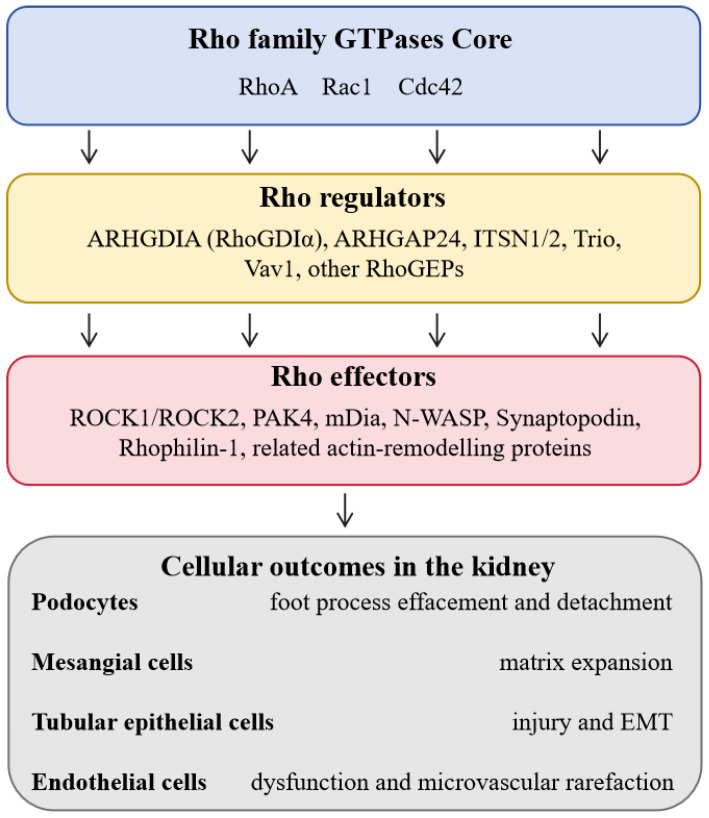
Rho GTPase regulatory network across major renal cell types. Schematic overview of how Rho family GTPases are organized and regulated in different renal cell types. Podocytes, mesangial cells, proximal tubular epithelial cells and glomerular endothelial cells all express the core Rho GTPases Cdc42, Rac1 and RhoA. Their activity is tightly controlled by guanine nucleotide exchange factors (GEFs), GTPase-activating proteins (GAPs) and GDP-dissociation inhibitors (GDIs), including ARHGDIA (RhoGDIα), ARHGAP24, ITSN1/2, Trio, Vav1 and other RhoGEFs/RhoGAPs. These regulators feed into a shared effector layer consisting of ROCK1/ROCK2, PAK4, mDia, N-WASP, Synaptopodin, Rhophilin-1 and related actin-remodeling proteins. Together, dysregulated Rho signaling drives key pathological outcomes in the kidney, including podocyte foot process effacement and detachment, mesangial matrix expansion, tubular epithelial injury and epithelial–mesenchymal transition (EMT), as well as endothelial dysfunction and microvascular rarefaction.

Dysregulation of Rho signaling is a key molecular event in the occurrence and development of various kidney diseases. Abnormal activation of Rho GTPases leads to podocyte foot process effacement and detachment, mesangial matrix expansion, tubular epithelial injury and EMT, as well as endothelial dysfunction and microvascular rarefaction in the kidney [5]. Therefore, studying the expression and regulatory mechanism of Rho family proteins in the kidney is of great significance for clarifying the molecular pathogenesis of kidney diseases, and targeting the Rho signaling pathway provides a potential novel therapeutic strategy for the treatment of acute and chronic kidney diseases. The Rho GTPase regulatory network across major renal cell types is detailed in Figure 2.

### 1.3. Structure and Function of ROCK

**Figure 3:**
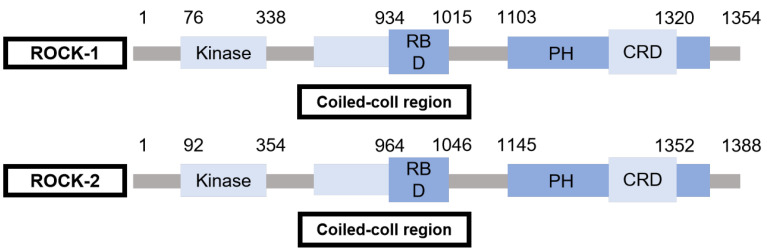
Structure of ROCK. The primary structure of ROCK: ROCK consists of an N-terminal catalytic domain, a middle helical domain containing the Rho-binding region (RBD), and a C-terminal cysteine-rich PH domain (CRD).

ROCK is a member of the serine/threonine protein kinase family and is currently the most well-researched downstream target effector molecule of Rho [13]. ROCK has two isomers, ROCKα/ROCK II and ROCKβ/ROCK I (or p160 ROCK). Both of these isomers are proteins with relatively large molecular weights (150–160 kD). ROCK I and ROCK II have 65% homology in the whole amino acid sequence and 92% similarity in catalytic domain [14]. They are widely distributed in most organs. ROCK I mainly exists in kidney tissue, while ROCK II is highly expressed in heart and brain [15]. ROCK is composed of N-terminal kinase domain, a helical domain containing Rho-binding region (RBD) in the middle and a cysteine-rich PH domain (CRD) at the C-terminal. ROCK receives the activation signal transmitted by Rho, activates by phosphorylation of multiple amino acid sites, and mediates a series of downstream phosphorylation/dephosphorylation reactions, which ultimately causes changes in the polymerization state of cellular actin, and promotes the formation of stress fibers and localization, thus affecting cell chemotaxis, adhesion, sensitivity to calcium ions, and cell cycle progression [16]. Structure of ROCK is shown in Figure 3.

**Figure 4:**
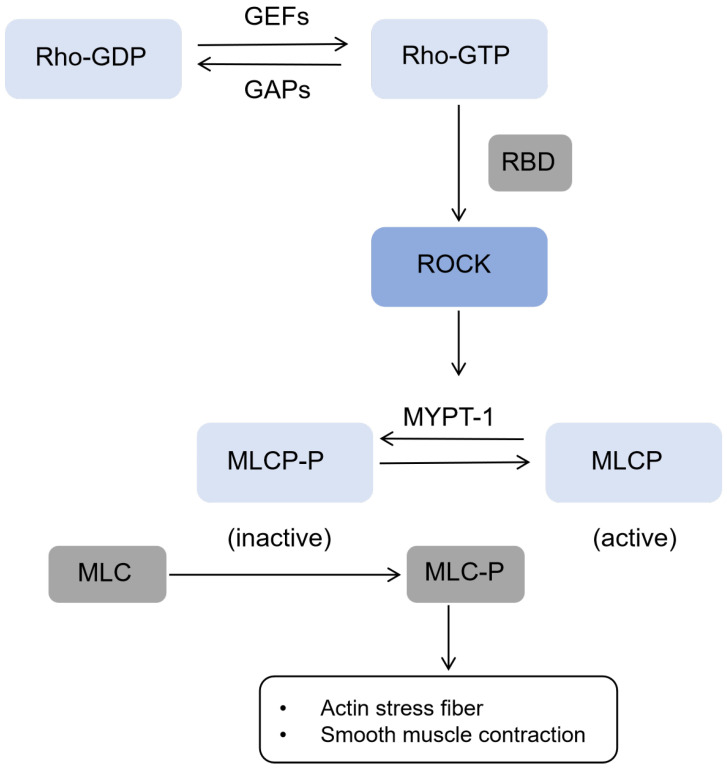
Downstream signaling of ROCK. The Rho/ROCK signaling pathway to MLCP/MLC: activated Rho GTPases bind to RBD and activate ROCK; activated ROCK phosphorylates MYPT1 (the regulatory subunit of MLCP), leading to MLCP inactivation; inactive MLCP loses the ability to dephosphorylate MLC, resulting in the accumulation of phosphorylated MLC, actin stress fiber formation and smooth muscle contraction. ROCK: Rho-associated coiled coil forming protein kinase; RBD: Rho-binding region; MLCP: myosin light chain phosphatase; MYPT1: myosin phosphate target subunit 1; MLC: myosin light chain; GDP, guanosine diphosphate; GTP, guanosine triphosphate; GEF, guanine nucleotide exchange factor; GAP, GTPase activating protein.

One of the main substrates of ROCK is myosin light chain phosphatase (MLCP), which is composed of three subunits, including myosin binding subunit (MYPT1) and phosphatase catalytic subunit. Activated ROCK phosphorylates MYPT1, thereby inactivating MLCP itself and losing its ability to catalyze the dephosphorylation of MLC. The level of phosphorylated MLC in the cytoplasm and the activity of myosin ATPase increased, resulting in the contraction of smooth muscle and the increase in myosin/myosin cross-linking, so as to promote the polymerization of actin microfilament skeleton [17]. Rho/ROCK signaling pathway controls the polymerization of cytoskeleton through cascade phosphorylation/dephosphorylation reaction, and affects the biological behavior of cells, such as adhesion between cells or extracellular matrix, cell migration, and cell phenotypic transformation [18]. Downstream signaling of ROCK are shown in Figure 4.

**Figure 5:**
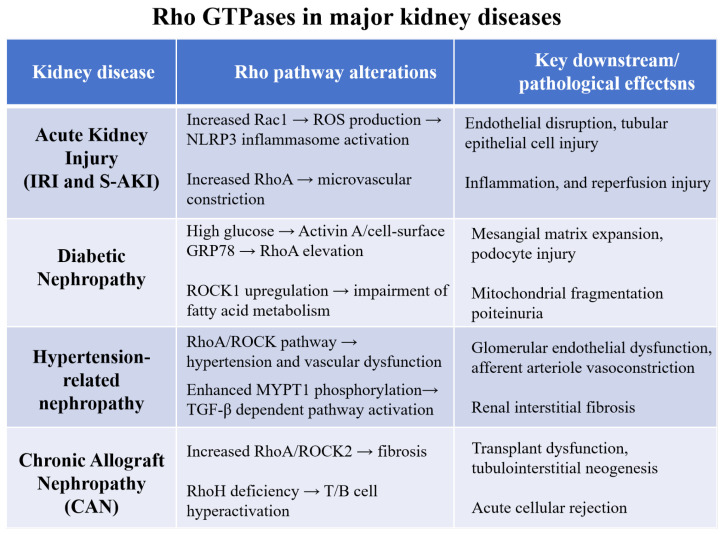
Rho GTPase pathway alterations across major kidney diseases. In acute kidney injury (IRI and S-AKI): increased Rac1 drives ROS production and NLRP3 inflammasome activation, leading to endothelial disruption and tubular epithelial cell injury; elevated RhoA promotes microvascular constriction, exacerbating inflammation and reperfusion injury. In diabetic nephropathy: high glucose induces RhoA elevation via Activin A/cell-surface GRP78, resulting in mesangial matrix expansion and podocyte injury; ROCK1 upregulation impairs fatty acid metabolism, triggering mitochondrial fragmentation and proteinuria. In hypertension-related nephropathy: the RhoA/ROCK pathway mediates hypertension and vascular dysfunction, contributing to glomerular endothelial dysfunction and afferent arteriole vasoconstriction; enhanced MYPT1 phosphorylation activates TGF-β-dependent pathways, driving renal interstitial fibrosis. In chronic allograft nephropathy (CAN): increased RhoA/ROCK2 promotes fibrosis, leading to transplant dysfunction and tubulointerstitial neogenesis; RhoH deficiency causes T/B cell hyperactivation, resulting in acute cellular rejection; IRI, ischemia-reperfusion injury; S-AKI, sepsis-associated acute kidney injury; ROS, reactive oxygen species.

At present, many ROCK inhibitors have been discovered; however, they are basically small molecule inhibitors [19]. According to their structural characteristics, they can be roughly divided into the following four categories: isoquinolines, 4-aminopyridines, indazoles, amides and ureas. The most common ROCK inhibitors are isoquinoline with remarkable structural feature of the sulfonyl-linked isoquinoline and high piperazine ring structural units [20]. Fasudil is the first developed and the only generally available ROCK inhibitor in Japan [21]. It is an effective drug against vasospasm and clinically applicable to ischemic cerebrovascular diseases. Fasudil is considered to be with the strongest inhibitory effect on ROCK so far. Therefore, it is often used as a reference drug in the study of ROCK in vitro [22]. The second category is 4-aminopyridines containing a 4-aminopyridine parent nucleus. Y-27632 is an outstanding representative among them. Because of its moderate inhibitory effect on ROCK, it is widely used in biological and pharmacological research [23,24,25]. The third category is indazoles which usually take 5-alkoxy-1h or 5-aminoindazole as the skeleton and then synthesize a variety of effective ROCK inhibitors. The results showed that aminoquinazoline, benzotetrahydropyrrole and pyrimidinyl aniline and other groups could be linked to the mother nucleus to obtain efficient ROCK inhibition [26]. The fourth category is amides and ureas. Currently, there are relatively few reports on this kind of mother nucleus structure, but many ROCK inhibitors with urea as pharmacodynamic group have been found [27]. The major alterations of the Rho/ROCK pathway in different kidney diseases are summarized in Figure 5.

The Rho/ROCK signaling pathway is a key regulator of cytoskeletal reorganization, and its abnormal activation is closely related to renal cell injury and fibrosis. The following sections of this review will elaborate on the specific mechanisms of the Rho/ROCK signaling pathway in acute kidney injury, diabetic nephropathy, hypertension-related nephropathy and chronic allograft nephropathy, and summarize the research progress of ROCK inhibitors in the treatment of these kidney diseases.

## 2. Acute Kidney Injury (AKI)

AKI refers to acute renal impairment, including acute mild renal insufficiency [28]. More and more evidences have shown that AKI significantly increases the long-term risk of developing chronic kidney disease (CKD) and directly leads to end-stage renal disease (ESRD) [29]. AKI occurs in approximately 5% of hospitalized patients and up to 40–60% of patients in the intensive care unit [30]. In addition, the incidence of AKI is increasing over the years [31].

### 2.1. Ischemic AKI

In adults, ischemia is still the main cause of acute renal failure [32]. Ischemic injury is characterized by loss of cell polarity and the release of proximal tubule epithelial cells due to cytoskeleton reorganization. Hypoxia and various renal injuries induce renal cell damage by regulating the activity of G proteins, including heterotrimeric G proteins and small G proteins (Rho GTPases). Ischemia/hypoxia activates the Gq/Gi family of heterotrimeric G proteins in renal microvascular endothelial cells and tubular epithelial cells, which further promotes the activation of RhoA and Rac1 GTPases. The cross-talk between heterotrimeric G proteins and Rho GTPases is a key molecular mechanism mediating renal microvascular constriction, oxidative stress and inflammatory infiltration in AKI, and inhibiting this G protein-related signaling cascade can effectively alleviate ischemic renal injury.

Rho GTPases protein and mRNA level were both up-regulated by ischemia in proximal tubule fractions. During reperfusion, the expression of Rac1 increased in soluble fractions [33,34]. ROCK1 protein level was also raised in the kidney after renal ischemia–reperfusion injury (IRI). Treatment with Y-27632 (a ROCK1 inhibitor) reduced the deterioration of renal function and renal tubular damage in mice after IRI. In HK-2 cells exposed to antimycin A, the expression of ROCK1 was activated, resulting in the up-regulation of caspase-3 and pro-inflammatory molecules, which was eliminated by Y-27632 [23].

Y-27632 treatment notably influenced the changes in hemodynamics in IRI model. Study has shown that Y-27632 increased renal blood flow in vivo and improved endothelium-dependent vasodilation in vitro, which might be mediated in part by reducing reactive oxygen species (ROS). The vasodilatory effect was independent of cyclooxygenase (COX) and the resulting vasoconstrictive prostaglandins [35]. Another study applied fluorescent microspheres to measure renal blood flow, and the results showed that ischemic/reperfusion (I/R) caused a 62% reduction in renal blood flow, while the decrease in blood flow in the Y-27632 treatment group was alleviated. In addition, after I/R, the renal interlobar artery displayed a decrease in phosphorylated endothelial nitric oxide synthase (eNOS) and phosphoprotein (a marker of bioactive NO) stimulated by vasodilators, which was also weakened by Y-27632 in vivo. These results suggest that inhibition of Rho kinase in IRI model can maintain renal blood flow by improving eNOS function [36]. At the same time, study has demonstrated that Y-27632 improved the accumulation of microvascular leukocytes in the early stage of renal cortex and medulla after IRI [25,37].

### 2.2. Toxic-Induced AKI

Toxic-induced AKI is caused by nephrotoxic drugs and contrast agents, and the Rho/ROCK pathway is a key mediator of toxic renal tubular epithelial cell injury and barrier damage. Clinically, cyclosporine A (CsA) and sirolimus (SRL) have a strong immunosuppressive effect, which also cause nephrotoxicity. The study found that the combination of CsA and SRL activated RhoA [38], while Y-27632 treatment prevented CsA and SRL-induced cofilin phosphorylation and actin remodeling, reduced the increase in transepithelial resistance (TER), and deregulated claudin-7 protein level. It can be seen that the Rho/ROCK pathway is involved in mediating CsA and SRL-induced cytoskeletal rearrangement and changes in TER through claudin-7 [39,40].

Doxorubicin (DOX), an anthracycline antibiotic, is one of the most effective anticancer drugs in clinic. It is widely used in the treatment of various human tumors, such as neurofibromatosis, liver cancer and breast cancer [41,42,43]. However, the use of DOX is greatly limited due to its toxic effects in multiple organs and tissues [44]. Available laboratory evidence shows that DOX can induce nephropathy, including increased glomerular capillary permeability and glomerular atrophy, as well as the accumulation of DOX in glomerulus [45]. In a mouse model, administration of fasudil significantly relieved DOX-induced kidney damage, inhibited cell apoptosis and senescence, and improved redox imbalance and DNA damage [46]. However, the specific regulatory mechanism and clinical research are still lacking.

Fasudil is currently the only ROCK inhibitor widely used in clinical practice. A large number of studies have shown that it has a protective effect on AKI caused by different reasons [47]. Fasudil relieved cisplatin-induced histological injury, improved renal function and reduced leukocyte infiltration in vivo. In vitro, fasudil played a protective role on damaged tubules, which depressed T cell apoptosis and cytokine production, and lessened proximal tubular cell apoptosis and tubular injury. Thus, inhibition of Rho kinase may be a therapeutic strategy to prevent cisplatin-induced AKI [48]. Metabolomic analysis of mice kidney tissue by ultra-performance liquid chromatography/quadrupole time-of-flight mass spectrometry showed that there were significant differences in metabolites both between the control group and cisplatin group and between cisplatin group and fasudil intervention group. These differential metabolites mainly focused on lipid and amino acid metabolism. It is suggested that fasudil-mitigated cisplatin induced AKI by interfering with related metabolism [49].

Based on IRI-induced AKI in male Sprague Dawley rats, compared with sham-treated group, hydroxyfasudil-treated group had decreased proteinuria and polyuria, and elevated urine osmotic pressure. In addition, renal perfusion (assessed by 18 fluorine positron emission tomography (PET)), creatinine and urine clearances were significantly improved. Endothelial leakage and kidney inflammation were also reduced [50]. With the development and popularization of cardiac diagnosis and treatment interventions, the impact of radiographic contrast agents has increased markedly. The use of radiographic contrast agents elevates the incidence and mortality of contrast induced acute renal injury (CI-AKI). Research showed that fasudil treatment improved the medullary damage induced by contrast medium, restored renal function, inhibited renal tubular cell apoptosis, and alleviated redox imbalance and DNA damage [51].

### 2.3. Sepsis-Associated AKI

**Figure 6:**
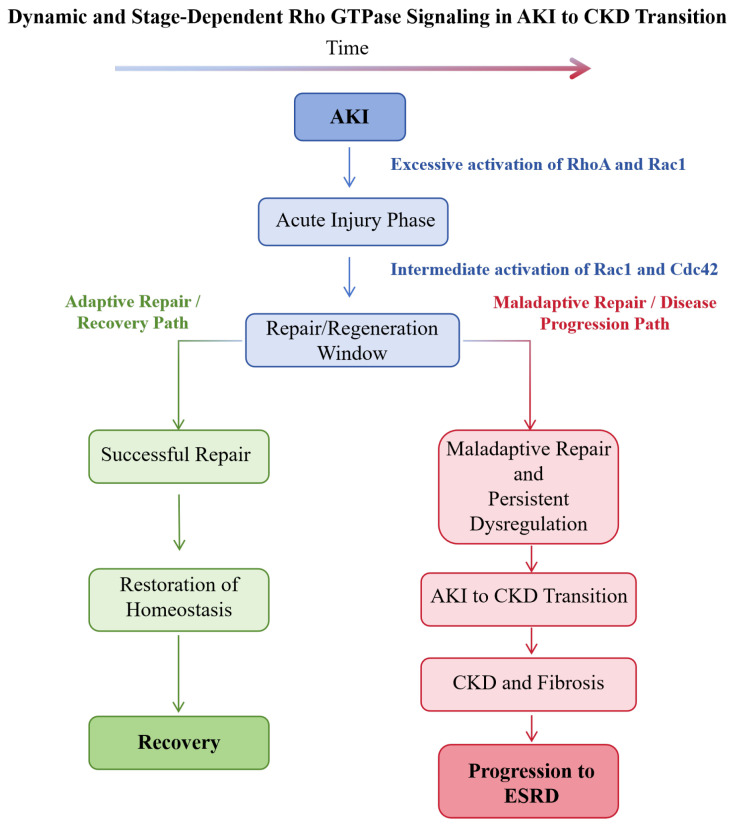
The dual role of Rho GTPases across the AKI-to-CKD continuum. Schematic illustration showing the dynamic and stage-dependent functions of Rho GTPases during the transition from AKI to CKD. In the early injury phase, excessive activation of RhoA and Rac1 contributes to endothelial contraction, reduced renal blood flow, oxidative stress, NLRP3 inflammasome activation and tubular epithelial cell injury. During the repair/regeneration window, intermediate activation of Rac1 and Cdc42 supports cytoskeletal remodeling, polarity restoration and tubular epithelial regeneration. Failure to resolve the acute injury or persistent dysregulation of RhoA/ROCK2 signaling leads to maladaptive repair, characterized by fibroblast activation, extracellular matrix accumulation, microvascular rarefaction and progressive renal fibrosis. This stage-dependent “double-edged sword” behavior highlights the need for temporally precise modulation of Rho GTPase signaling in therapeutic interventions. AKI, acute kidney injury; CKD, chronic kidney disease; ESRD, end-stage renal disease.

Sepsis-associated AKI is the most common type of AKI in the intensive care unit, and its pathogenesis is associated with systemic inflammation, microcirculatory disorder and renal hypoperfusion. The Rho/ROCK pathway is overactivated in sepsis-associated AKI, and Rac1 activation promotes NLRP3 inflammasome activation and ROS production in renal cells, exacerbating inflammatory injury and tubular cell apoptosis [41]. Fasudil protects against sepsis-induced AKI in rats by suppressing the STAT-3/NLRP3 pathway via ROCK inhibition, which reduces renal inflammatory infiltration and improves renal function [47]. The dual role of Rho GTPases in the AKI-to-CKD continuum is illustrated in Figure 6.

## 3. Glomerular Diseases

### 3.1. Diabetic Nephropathy (DN)

Diabetic nephropathy (DN) is a major microvascular complication of type 1 and type 2 diabetes mellitus and is the leading cause of end-stage renal disease (ESRD) worldwide. The clinical features of DN include progressive albuminuria, glomerular filtration rate (GFR) decline, glomerulosclerosis and tubulointerstitial fibrosis. DN has a complex pathogenesis, which is affected by multiple factors including genetic background, metabolic disorders and hemodynamic abnormalities. Hyperglycemia is the initiating factor of DN, and persistent high glucose induces renal cell injury through multiple molecular pathways, ultimately leading to irreversible renal structural and functional damage. At present, the clinical treatment of DN is mainly based on blood glucose and blood pressure control, but these strategies can only delay the progression of the disease and cannot reverse renal fibrosis, so it is urgent to explore new therapeutic targets and drugs for DN.

#### 3.1.1. ROCK Inhibitors in the Treatment of DN

ROCK inhibitors represented by fasudil and Y-27632 exert nephroprotective effects on DN through direct effects on glomerular cells and indirect effects on extra-renal cells, and the reduction in proteinuria is the core therapeutic effect of these drugs.

##### Direct Effects on Glomerular Cells

The glomerulus consists of the following three major cell types: endothelial cells, mesangial cells and podocytes, and ROCK inhibitors directly target these cells to protect glomerular structure and function. For podocytes, Fasudil and Y-27632 inhibit Rho/ROCK pathway activation, reduce podocyte apoptosis, prevent mitochondrial fission and maintain podocyte foot process integrity, thereby reducing albuminuria [10]. For mesangial cells, ROCK inhibitors inhibit high glucose-induced mesangial cell proliferation and mesangial matrix expansion by downregulating the AP-1 and HIF-1α pathways, alleviating glomerulosclerosis. For glomerular endothelial cells, ROCK inhibitors improve endothelial dysfunction by inhibiting oxidative stress and NOX4 expression, maintaining glomerular microvascular integrity [52,53,54].

##### Indirect Effects on Extra-Renal Cells

ROCK inhibitors exert indirect nephroprotective effects by regulating the function of extra-renal cells including hepatocytes and cardiac cells [55,56,57]. In type 2 diabetes, fatty liver is closely associated with the progression of DN, and the Rho/ROCK pathway is overactivated in the liver of diabetic mice [58,59]. ROCK inhibitors inhibit hepatic Rho/ROCK activation, improve fatty acid metabolism disorders and reduce insulin resistance, thereby indirectly alleviating renal injury [60,61]. In diabetic cardiomyopathy, Rho/ROCK pathway activation mediates cardiac fibrosis and heart failure, which exacerbates renal hemodynamic abnormalities. ROCK inhibitors improve cardiac function by inhibiting cardiac Rho/ROCK activation, indirectly reducing renal perfusion damage [13].

##### Reduction in Proteinuria: Core Therapeutic Effect

Reducing proteinuria is the main therapeutic strategy for DN, as persistent albuminuria is a key predictor of DN progression to ESRD. ROCK inhibitors exert a significant anti-proteinuria effect in DN models: fasudil reduces urinary albumin excretion and 8-hydroxydeoxyguanosine levels in diabetic rats without affecting blood glucose and blood pressure, and Y-27632 alleviates proteinuria by improving renal hemodynamics and maintaining glomerular filtration barrier integrity [62,63,64,65]. The anti-proteinuria effect of ROCK inhibitors is mainly mediated by protecting podocyte function and inhibiting glomerulosclerosis, which is independent of blood pressure control, suggesting that ROCK inhibitors are a potential therapeutic drug for refractory proteinuria in DN.

#### 3.1.2. Glomerulosclerosis Induced by Rho/ROCK Pathway Activation

Fasudil has been shown to have diverse effects in DN. Study has shown that fasudil treatment reduced renal ROCK activity, improved the changes in kidney structure induced by diabetes, and had a moderate anti-proteinuria effect, but there was no change in blood pressure. Late intervention with fasudil alleviated glomerular sclerosis, without effect on proteinuria [66]. Another study found that fasudil had no effect on blood glucose and blood pressure in diabetic rats. However, it reduced the excretion of albumin and 8-hydroxydeoxyguanosine in urine. Meanwhile, the upregulation of TGF-β, connective tissue growth factor (CTGF) and NADPH-oxidase 4 (NOX4) mRNA in renal cortex of diabetic rats were inhibited by fasudil [67]. In recent years, some new mechanisms of fasudil in the treatment of DN have been discovered. Xie et al. demonstrated that fasudil postponed the progression of DN possibly by inducing M2 macrophage polarization, reducing M1 macrophage polarization and inflammation [68]. Moreover, fasudil treatment did not improve diabetes-related metabolic abnormalities, but significantly improved renal artery and endothelial dysfunction, and reduced mesangial matrix expansion in the renal cortex. Mechanistically, superoxide production in the internal renal artery and NOX4 members of NADPH oxidase in the renal cortex were also blocked by Rho kinase inhibitors. Therefore, ROCK is involved in endothelial dysfunction in type 1 diabetes mellitus by enhancing oxidative stress [69].

#### 3.1.3. Podocyte Apoptosis Mediated by Rho/ROCK Signaling

In addition to Y-27632 and fasudil, possible inhibitors of Rho-related pathway in the kidney of DN have also been found recently [70]. Study has reported that activin inhibition with follistatin and neutralization of cell-surface GRP78 using a specific antibody inhibited RhoA activation in mesangial cells and in diabetic kidneys, which need to be further evaluated for their efficacy in inhibiting the progression of DKD [71].

Early diabetic nephropathy is characterized by abnormal proliferation of glomerular mesangial cells (MCs), which secrete a large number of mesangial matrixes, gradually blocking capillary vessels and glomerulosclerosis. Glomerular MCs play an important role in glomerular injury and repair. Exposure of MCs to high glucose (HG) resulted in increased expression of membrane associated Ras and Rho GTPase proteins, while simvastatin treatment reversed HG induced Ras and Rho membrane translocation [72]. Meanwhile, RhoA siRNA and dominant-negative RhoA significantly reduced the upregulation of fibronectin by HG in MCs mediated by activator protein-1 (AP-1) activation [73]. Interference with Src/caveolin-1/RhoA signaling may also be involved in HG-induced FN upregulation in MCs [74]. Hypoxia-inducible factor -1α (HIF-1α) is a key regulator of renal sclerosis in diabetic patients. Study has shown that the pharmacological and genetic inhibition of Rho kinase promoted the degradation of proteasomal HIF-1α, and then inhibits the expression of HIF-1-dependent profibrotic genes by upregulating prolyl hydroxylase 2 [75]. The activation of NF-κB plays a pivotal role in the pathogenesis of DN. The increase in F-actin induced by RhoA/ROCK signal promoted the association between zonula occludens-1 (ZO-1) and connexin43 (Cx43), which might trigger the endocytosis of Cx43, thus facilitating the NF-κB activation in HG-treated MCs [76].

Evidence shows that podocyte apoptosis is a key event in the development of DN [77]. Renal biopsy reveals that the podocyte density decreases in DN patients, and the number of podocytes begin to decrease before the onset of microalbuminuria. With the decrease in podocyte, DN progresses rapidly, indicating that podocyte apoptosis is also one of the pathological changes in early DN [10,78]. Study showed that GTP-binding Rac1, Cdc42, Rho A, and ROCK were upregulated in glucose-stimulated podocytes and diabetic kidneys [79]. In Rac1 gene knockout (KO) group mice, the urinary albumin/creatinine ratio (ACR) was significantly higher than that in WT group. Electron microscopy analysis showed that the Rac1 KO groups had higher foot loss rate compared with WT groups, at the same time, the number of WT1 positive cells in STZ/KO group was significantly lower than that in the other three groups. Hence, podocyte-specific Rac1 deletion leads to podocyte morphological changes [80]. By generating two diabetic mouse models with targeted deletion of ROCK1 and an inducible podocyte-specific knockin mouse expressing a constitutively active (cA) mutant of ROCK1, results showed that ROCK1 deletion in diabetic mice prevented mitochondrial fission, while podocyte-specific cA-ROCK1 mice displayed increased mitochondrial fission. The mechanism may be through the phosphorylation dynamin-related protein-1 (Drp1) at serine 600 residue [81,82]. After treatment with the Rho kinase inhibitor fasudil in db/db mice, it was found that proteinuria and urinary excretion of renin were reduced as well as the decrease in podocyte apoptosis and Jag1 expression [83,84]. At the same time, fasudil also blocked the formation of microparticles in podocytes induced by HG [85].

### 3.2. Hypertension-Related Nephropathy

Hypertensive nephropathy is the damage of renal structure and function caused by essential hypertension. High blood pressure increases the blood pressure in vessels, resulting in protein leakage into the urine, which will damage the filter screen system of the kidney. If hypertension is poorly controlled for a long time and the structural damage caused is difficult to reverse, renal function will gradually brock down, develop into CKD, and may eventually develop into uremia [86].

In a rodent model of salt-sensitive hypertension, high salt load activated Rac1 in the kidney, resulting in increased blood pressure and renal injury through a mineralocorticoid receptor (MR)-dependent pathway [87,88,89]. Recent studies have shown that aldosterone played an important role in the pathogenesis of renal injury [90,91]. Rats receiving aldosterone infusion showed hypertension and severe renal injury [92]. Fasudil treatment did not change blood pressure, but significantly improved proteinuria and renal injury in aldosterone infused rats. Moreover, fasudil inhibited myosin phosphate target subunit-1 (MYPT1) phosphorylation and significantly depressed the activation of TGF-β-dependent pathway [93]. In addition, Y-27632 treatment also restrained aldosterone induced epithelial-to-mesenchymal transition (EMT) and extracellular matirx (ECM) accumulation in the kidney [94]. Another study demonstrated ROCK pathway was involved in angiotensin II (AngII)-induced renal injury by regulating pro-inflammatory and pro-fibrotic mediators [95]. In this model, Rho/ROCK pathway also played a major role in mediating basal and AngII-induced tone of afferent, but not efferent, arterioles [96]. eNOS-deficient mice showed increased renal vascular tension and systemic hypertension. Compared with WT mice, eNOS KO mice had higher Rho kinase activity. However, after treatment with Y-27632, renal vasodilation increased and blood pressure decreased, suggesting the important role of ROCK signaling pathway in hypertension and vascular dysfunction in eNOS KO mice [97]. Winaver et al. proposed that Rho kinase-dependent pathway was involved in the pathological process of renal vasoconstriction and myocardial hypertrophy in heart failure rats with volume overload. Selective blocking of the signaling pathway may be considered as an effective tool to improve renal perfusion and reduce myocardial hypertrophy in patients with heart failure [98].

Fasudil and Y-27632 exert nephroprotective effects on hypertension-related nephropathy through direct protective effects on glomerular cells and indirect anti-hypertensive effects, and the reduction in proteinuria is the key therapeutic endpoint of these drugs. The main pathophysiology of hypertension-related nephropathy is focal segmental glomerulosclerosis (FSGS), and ROCK inhibitors directly target glomerular endothelial cells, mesangial cells and podocytes: inhibit Rho/ROCK pathway activation in podocytes to maintain foot process integrity, reduce mesangial matrix expansion and alleviate FSGS, and improve glomerular endothelial dysfunction, thereby reducing proteinuria. The indirect anti-hypertensive effect of ROCK inhibitors is mainly mediated by inhibiting the Rho/ROCK pathway in vascular smooth muscle cells, reducing renal afferent arteriole vasoconstriction and improving renal microvascular tone, which further alleviates renal hemodynamic damage. Notably, fasudil improves proteinuria and renal injury in aldosterone-infused rats without changing blood pressure, indicating that the direct glomerular protective effect of ROCK inhibitors is independent of their blood pressure-lowering effect, and this feature makes them a potential adjuvant drug for hypertension-related nephropathy with refractory proteinuria.

### 3.3. Chronic Allograft Nephropathy (CAN)

CAN is the main cause of late functional loss of transplanted kidney and an important factor influencing the long-term survival of transplanted kidney. It refers to the progressive decline of renal function after allogeneic kidney transplantation for several months, especially one or several years, and finally develop to renal failure and recovery of dialysis. Histologically, the transplanted kidney shows interstitial fibrosis, thickening and shrinkage of glomerular basement membrane, increase in mesangial matrix, arteriosclerosis, hyaline degeneration of arterioles, thickening of fibrous intima of arteries, stratification and fracture of capillary basement membrane, etc.

It was found that RhoA and ROCK-1 mRNA and protein level increased in mesangial cells and tubular cells, gradually accompanying with the progress of CAN. Meanwhile, RhoA/ROCK-1 mRNA was negatively correlated with Banff score of chronic rejection of transplanted kidney [99,100]. The specific inhibition of Rho activity by Y-27632 had a positive effect on blocking renal interstitial inflammation and fibrosis, so as to effectively delay the development of CAN [101,102]. Y-27632 coupled with lysozyme (Y-27632-lysozyme) can specifically release its drug in proximal tubular cells. After allogeneic transplantation, Y-27632-lysozyme markedly reduced the accumulation of mesenchymal macrophages on the 4th day after transplantation. Compared with the control group, the interstitial lymphangiogenesis induced by the allograft was also depressed, suggesting that the inhibition of ROCK decreased renal inflammation and renal lymphangiogenesis, which might be a valuable future treatment for renal transplantation [101].

RhoH deficiency is an important molecular abnormality in the progression of CAN. RhoH is a negative regulator of T and B cell activation, and RhoH deficiency in renal allografts leads to hyperactivation of T and B lymphocytes, which exacerbates acute cellular rejection and promotes the development of interstitial fibrosis and tubular atrophy. The interaction between RhoH deficiency and RhoA/ROCK pathway activation in CAN remains to be further studied, and targeting RhoH may be a new potential strategy for the treatment of allograft rejection.

## 4. Other Kidney Diseases

Nephrolithiasis is a common kidney disease and one of the major causes of chronic renal insufficiency. In a glyoxylate-induced mouse model of kidney calcium oxalate crystal deposition, fasudil decreased the formation and deposition of renal calcium crystals and slowed down the formation of renal fibers caused by calcium crystal deposition. The possible mechanism might be related to Rho/ROCK signal transduction and the regulation of EMT [103].

Mice on a high-fat diet not only developed obesity, but also displayed renal histological changes, including glomerular hypercellularity and mesangial matrix accumulation, which paralleled the increase in proteinuria [104]. Rho kinase activation and upregulation of inflammatory chemokines were found in the kidneys of mice fed with a high-fat diet [104]. SHRSP.Z-Leprfa/IzmDmcr (SHRSP fatty) rats create a new animal model of metabolic syndrome, accompanied by renal insufficiency, podocyte damage, EMT occurrence. It was found that ROCK activation also existed in this model [105]. The above studies show that the abnormality of lipid metabolism leads to kidney injury and activates Rho-related signaling pathway; however, whether Rho-related signaling pathway plays a key role in lipid-induced kidney injury still needs to be further studied.

## 5. Epithelial-to-Mesenchymal Transition (EMT) in Kidney Diseases

EMT is a key cellular process in the progression of CKD, which refers to the transformation of renal tubular epithelial cells into mesenchymal cells, leading to extracellular matrix (ECM) accumulation and renal interstitial fibrosis. The prevention of EMT is one of the most important targeted strategies for inhibiting CKD progression, and the Rho/ROCK signaling pathway is a core regulator of renal EMT [5].

Rho/ROCK pathway activation induces EMT in renal tubular epithelial cells by regulating cytoskeletal reorganization and the expression of EMT-related transcription factors. In hypertension-related nephropathy, aldosterone activates the Rho/ROCK pathway and induces renal EMT and ECM accumulation, which is reversed by Y-27632 treatment [85]. In nephrolithiasis, calcium oxalate crystal deposition activates the Rho/ROCK pathway, promoting renal EMT and fibrogenesis, and fasudil treatment inhibits crystal-induced EMT and reduces renal fibrosis [103]. In obesity-related renal injury, high-fat diet induces ROCK activation in the kidney, which mediates podocyte damage and renal EMT, and ROCK inhibitors alleviate obesity-induced renal structural and functional damage [104].

The Rho/ROCK pathway mediates EMT in kidney diseases through multiple mechanisms, including the regulation of actin cytoskeleton, the activation of TGF-β/Smad signaling, and the production of ROS. Targeting the Rho/ROCK pathway to inhibit renal EMT is a promising strategy for the treatment of progressive CKD, and ROCK inhibitors have shown potential in preclinical studies to suppress EMT and reduce renal fibrosis.

## 6. Conclusions

The incidence of acute and chronic kidney diseases is increasing globally, and the lack of effective therapeutic strategies for irreversible renal fibrosis and ESRD remains a major clinical challenge. As key intracellular signaling molecules, Rho GTPases and their downstream effector ROCK play a pivotal role in the pathogenesis of various kidney diseases including AKI, DN, hypertension-related nephropathy and CAN. The Rho/ROCK signaling pathway modulates multiple pathological processes in the kidney, such as podocyte injury, mesangial matrix expansion, tubular EMT, renal microvascular dysfunction and inflammatory infiltration, and the dysregulation of this pathway is a common molecular feature of different kidney diseases. However, the activation pattern and regulatory mechanism of the Rho/ROCK pathway are disease-specific: for example, Rac1 overactivation mediates oxidative stress and inflammatory injury in AKI, while RhoA/ROCK1 activation is the main driver of podocyte apoptosis and glomerulosclerosis in DN.

ROCK inhibitors represented by fasudil and Y-27632 have shown promising nephroprotective effects in preclinical studies, which can alleviate renal injury by inhibiting the Rho/ROCK pathway, reducing proteinuria and suppressing renal fibrosis. Fasudil is the only clinically available ROCK inhibitor, and its protective effects on AKI, DN and contrast-induced renal injury have been confirmed in animal models, while Y-27632 is widely used in basic research and shows potential therapeutic value for renal allograft injury. However, the current research on ROCK inhibitors still has obvious limitations: most studies are limited to animal models, and there is a lack of large-sample, multicenter clinical trials to verify their efficacy and safety in kidney disease patients; in addition, the existing ROCK inhibitors have poor target selectivity, which may lead to off-target side effects.

In addition, the crosstalk between the Rho/ROCK pathway and other classical nephroprotective signaling pathways (e.g., RAAS, SGLT2) remains to be further elucidated, and the combined application of ROCK inhibitors with RAAS blockers or SGLT2 inhibitors may be a new direction for the treatment of progressive kidney diseases. Future research should focus on developing kidney-specific and isoform-selective ROCK inhibitors, clarifying the clinical therapeutic window of ROCK inhibitors, and carrying out high-quality clinical trials to validate the translational value of targeting the Rho/ROCK pathway for kidney disease treatment. In summary, the Rho/ROCK signaling pathway is a promising molecular target for nephroprotective therapy, and in-depth research on this pathway will provide new strategies for the prevention and treatment of acute and chronic kidney diseases.

## Data Availability

No new data were created or analyzed in this study.

## Abbreviations

The following abbreviations are used in this manuscript:

Rho GTPases	Rho family proteins
ROCK	Rho-associated coiled coil forming protein kinase
GTP	Guanosine triphosphate
RhoA	Ras homolog family member A
Rac1	Ras-related C3 botulinum toxin substrate 1
Cdc42	Cell division cycle 42
EMT	Epithelial to mesenchymal transition
G protein	Guanine nucleotide binding protein
GDP	Guanosine diphosphate
GEFs	Guanine nucleotide exchange factors
GAPs	GTPase activating proteins
GDIs	Guanine nucleotide dissociation inhibitors
LPA	Lysophosphatidic acid
RBD	Rho binding region
PH domain	Pleckstrin homology domain
MLCP	Myosin light chain phosphate
MYPT1	Myosin-binding subunit of MLCP
MLC	Myosin light chain
ATPase	Adenosine triphosphatase
AKI	Acute kidney injury
CKD	Chronic kidney disease
ESRD	End stage renal disease
IRI	Ischemia–reperfusion injury
ROS	Reactive oxygen species
COX	Cyclooxygenase
I/R	Ischemic/reperfusion
eNOS	Endothelial nitric oxide synthase
NO	Nitric oxide
CsA	Cyclosporine A
SRL	Sirolimus
TER	Transepithelial resistance
PET	Positron emission tomography
CI-AKI	Contrast-induced acute renal injury
DN	Diabetic nephropathy
PKC	Protein kinase C
RAAS	Renin–angiotensin–aldosterone system
TSC	Tuberous sclerosis complex
mTOR	Mammalian target of rapamycin
DKD	Diabetic kidney disease
STZ	Streptozotocin
WT	Wild type
TGF-β1	Transforming growth factor beta 1
CTGF	Connective tissue growth factor
NOX4	NADPH oxidase 4
FPCL	Fibroblast-populated collagen lattice
MCs	Mesangial cells
HG	High glucose
AP-1	Activator protein 1
HIF-1α	Hypoxia-inducible factor 1α
NF-κB	Nuclear factor kappa B
ZO-1	Zonula occludens 1
Cx43	Connexin43
KO	Knockout
ACR	Albumin/creatinine ratio
Drp1	Dynamin-related protein 1
MR	Mineralocorticoid receptor
AngII	Angiotensin II
MYPT1	Myosin phosphate target subunit 1
ECM	Extracellular matrix
CAN	Chronic allograft nephropathy
DOX	Doxorubicin
